# Erythromycin A dimethyl sulfoxide disolvate 1.43-hydrate

**DOI:** 10.1107/S1600536812005223

**Published:** 2012-02-17

**Authors:** Jürgen Brüning, Tanja K. Trepte, Jan W. Bats, Martin U. Schmidt

**Affiliations:** a Institute of Inorganic and Analytical Chemistry, University of Frankfurt, Max-von-Laue-Strasse 7, 60438 Frankfurt, Germany; b Institute of Organic Chemistry and Chemical Biology, University of Frankfurt, Max-von-Laue-Strasse 7, 60438 Frankfurt, Germany

## Abstract

The title compound, C_37_H_67_NO_13_·2C_2_H_6_OS·1.43H_2_O, is a macrolide anti­biotic with better solubility and better dermal penetration abilities than erythromycin A itself. The asymmetric unit of this form contains one erythromycin A mol­ecule, two dimethyl sulfoxide (DMSO) solvent mol­ecules, a fully occupied water mol­ecule and a partially occupied water mol­ecule with an occupancy factor of 0.432 (11). The 14-membered ring of the erythronolide fragment has a conformation which differs considerably from that in erythromycin A dihydrate [Stephenson, Stowell, Toma, Pfeiffer & Byrn (1997 ▶). *J. Pharm. Sci.* **86**, 1239–1244]. One of the two DMSO mol­ecules is disordered over two orientations; the orientation depends on the presence or absence of the second, partially occupied, water mol­ecule. In the crystal, erythromycin mol­ecules are connected by O—H⋯O hydrogen bonds involving the hy­droxy groups and the fully occupied water mol­ecule to form layers parallel to (010). These layers are connected along the *b*-axis direction only by a possible hydrogen-bonding contact involving the partially occupied water mol­ecule.

## Related literature

For a description of the title compound, see: Schmidt *et al.* (2011 ▶). For general background, see: Woodward *et al.* (1981 ▶). For crystallization experiments, see: Mirza *et al.* (2003 ▶). For related structures, see: Stephenson *et al.* (1997 ▶); Henry & Zhang (2007 ▶); Tian *et al.* (2009 ▶). For refinement details, see: Flack (1983 ▶); Spek (2009 ▶).
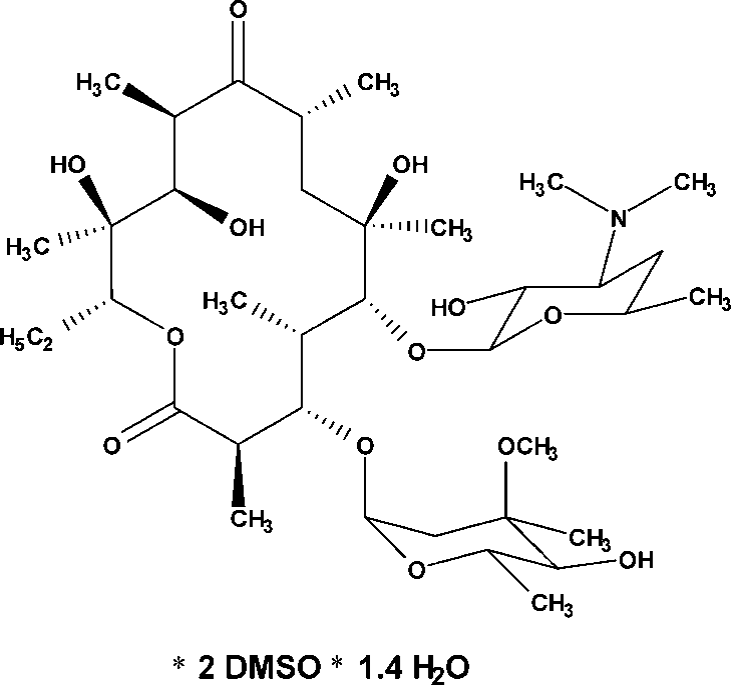


## Experimental

### 

#### Crystal data

C_37_H_67_NO_13_·2C_2_H_6_SO·1.43H_2_O
*M* *_r_* = 915.93
Monoclinic, 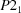

*a* = 11.1716 (7) Å
*b* = 19.4025 (12) Å
*c* = 12.0025 (7) Å
β = 106.245 (1)°
*V* = 2497.8 (3) Å^3^
*Z* = 2
Mo *K*α radiation
μ = 0.17 mm^−1^
*T* = 296 K
0.55 × 0.42 × 0.32 mm

#### Data collection

Siemens SMART 1K CCD diffractometer
Absorption correction: multi-scan (*SADABS*; Sheldrick, 2000 ▶) *T*_min_ = 0.870, *T*_max_ = 0.947
27453 measured reflections
10520 independent reflections
7109 reflections with *I* > 2σ(*I*)
*R*_int_ = 0.037

#### Refinement

*R*[*F* ^2^ > 2σ(*F* ^2^)] = 0.062
*wR*(*F* ^2^) = 0.142
*S* = 1.04
10520 reflections
579 parameters
23 restraints
H atoms treated by a mixture of independent and constrained refinement
Δρ_max_ = 0.37 e Å^−3^
Δρ_min_ = −0.34 e Å^−3^
Absolute structure: Flack (1983 ▶), with 4965 Friedel pairs
Flack parameter: 0.02 (9)

### 

Data collection: *SMART* (Siemens, 1995 ▶); cell refinement: *SAINT* (Siemens, 1995 ▶); data reduction: *SAINT*; program(s) used to solve structure: *SHELXS97* (Sheldrick, 2008 ▶); program(s) used to refine structure: *SHELXL97* (Sheldrick, 2008 ▶); molecular graphics: *SHELXTL* (Sheldrick, 2008 ▶); software used to prepare material for publication: *SHELXL97*.

## Supplementary Material

Crystal structure: contains datablock(s) I, global. DOI: 10.1107/S1600536812005223/sj5188sup1.cif

Structure factors: contains datablock(s) I. DOI: 10.1107/S1600536812005223/sj5188Isup2.hkl

Additional supplementary materials:  crystallographic information; 3D view; checkCIF report

## Figures and Tables

**Table 1:** Hydrogen-bond geometry (Å, °)

*D*—H⋯*A*	*D*—H	H⋯*A*	*D*⋯*A*	*D*—H⋯*A*
O6—H6*A*⋯O7*W*^i^	0.82	2.00	2.810 (4)	170
O7*W*—H7*C*⋯O26^ii^	0.85 (1)	2.07 (2)	2.888 (4)	163 (4)
O7*W*—H7*D*⋯N33	0.84 (1)	2.04 (2)	2.863 (4)	166 (5)
O11—H11*B*⋯O12	0.82	2.07	2.575 (4)	120
O12—H12*A*⋯O15	0.82	1.85	2.602 (8)	152
O12—H12*A*⋯O15′	0.82	2.04	2.667 (7)	134
O25—H25*B*⋯O11^iii^	0.82	2.04	2.838 (4)	166
O34—H34*B*⋯O14	0.82	1.94	2.747 (4)	170

Symmetry codes: (i) 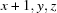; (ii) 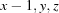; (iii) 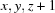.

**Fig. 1.**
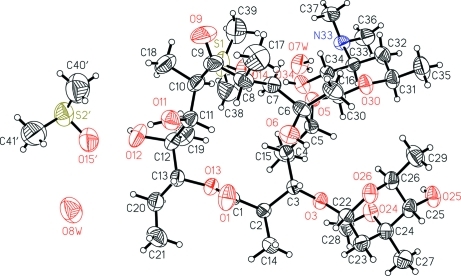
The asymmetric unit of (I) at 296 K with the numbering scheme of the atoms. Displacement ellipsoids are drawn at the 50% probability level and H atoms are shown as small spheres of arbitrary radii. Atoms belonging to the second orientation of the disordered DMSO solvate molecule have been omitted for clarity.

## supplementary crystallographic information

### Comment

Erythromycin is a macrolide antibiotic with a wide antimicrobial spectrum (Woodward *et al.*, 1981; Schmidt *et al.*, 2011). The dimethyl sulfoxide disolvate 1.4-hydrate of erythromycin A reported here may have potential as an active pharmaceutical ingredient (API), because it shows enhanced solubility and better dermal penetration abilities than erythromycin A itself (Schmidt *et al.*, 2011). The title compound can be used as an antibiotic, *e.g.* for defence against germs such as bacillus anthracis, streptococcaceae, bordetella, legionellaceae, and chlamydiaceae. It could also have a role in the treatment of infections, *e.g.* in the ear, nose, and throat regions, especially in the middle ear and the paranasal sinuses. It could be used to treat infections in the deep respiratory passages against *e.g.* bronchitis, pneumonia, pertussis, and infection of the conjunctiva. Other uses include the treatment of erysipelas, diphtheria, severe forms of acne vulgaris, inflammation of the skin and urethra, and dysfunction of motility and excretion. It could treat inflammation of the gastro and intestinal tract as well as inflammation of the pharynx, *e.g.* pharyngitis, tonsillitis, or scarlatina, syphilis and actinomycosis. It is a potential prophylactic against rheumatic fever after infection, especially in case of penicillin allergy. The compound may also be applied to comparable diseases in veterinary medicine as well. The crystalline forms of a number of other solvates of erythromycin A have been characterized by Stephenson *et al.* (1997), Mirza *et al.* (2003) and Henry & Zhang (2007).

The asymmetric unit of the dimethyl sulfoxide disolvate 1.4-hydrate of erythromycin A, (I, Fig. 1), C_37_H_67_NO_13_.2(C_2_H_6_OS).1.4(H_2_O), at 296 K contains one erythromycin A molecule, two DMSO solvate molecules, a fully occupied water molecule and a partially occupied water molecule with an occupancy factor of 0.432 (11). The 14-membered ring has a conformation which differs considerably from that in the crystal structure of erythromycin A dihydrate (Stephenson *et al.*, 1997). For the two structures, the torsion angles in the fragment C10–C11–C12–C13–O13–C1–C2–C3–C4–C5–C6 are rather similar, but torsion angles in the fragment C6–C7–C8–C9–C10 are considerably different. The six-membered rings of the side-chains have the usual chair conformation. The partially occupied water molecule lies too close to the methyl group C41 of the disordered DMSO molecule [distance O8W···C41 2.439 (15) Å]. Thus, if the water molecule is present, the disordered DMSO molecule must have the orientation defined by positions S2', C40', C41', O15'. A search for possible solvent accessible voids with program *PLATON* (Spek, 2009) reveals that no voids are present in the unit cell if the disordered DMSO molecule has the orientation defined by positions S2, O15, C40, C41, but that a void of 16 Å^3^ at the position of atom O8W occurs when this DMSO molecule has the orientation defined by positions S2', O15', C40', C41'. No H atoms were located at the partially occupied water molecule (O8W). However, O8W shows possible hydrogen bonding contacts with atom O15' of the disordered DMSO molecule [O8W···O15' = 3.146 (11) Å] and with atom O14 of the ordered DMSO molecule [O8W···O14^i^ = 2.971 (10) Å; i: 1 - *x*, 1/2 + *y*, 1 - *z*]. The O15'—O8W—O14^i^ angle of 139.8 (4)°, however, appears too large for both hydrogen bonds to occur simultaneously. A partially occupied water molecule has also been observed in the crystal structure of the closely related compound clarithromycin (Tian *et al.*, 2009). The fully occupied water molecule (O7W, H7C, H7D) accepts a hydrogen bond from the hydroxy group O6—H6A and acts as a donor for two hydrogen bonds to two symmetry-related erythromycin molecules (Table 1).

The erythromycin molecule shows one intramolecular O—H···O hydrogen bond. The remaining hydroxy groups are involved in intermolecular hydrogen bonds. One of them links the erythromycin molecules along the *c*-axis direction. The other three hydroxy groups are directed towards the two DMSO solvate molecules and towards water molecule O7W. The latter water molecule links the erythromycin molecules along the *a*-axis direction. Thus, the structure contains hydrogen bonded layers parallel to the (010) plane. The only intermolecular contact between adjacent layers along the *b*-axis direction is the before mentioned possible O8W···O14^i^ hydrogen bond, involving the partially occupied water molecule.

A second crystal of the title compound was measured at 177 K. The resulting crystal structure was similar to the structure determined at room temperature. The occupancy factor of the partially occupied water molecule refined to 0.400 (7). No phase transition was observed on cooling of the crystal from room temperature to 177 K.

### Experimental

Erythromycin A was recrystallized from a dimethyl sulfoxide/water mixture. The crystal used for the data collection was sealed in a glass capillary tube with a drop of mother liquor.

The compound was also characterized by its X-ray powder pattern which shows the following characteristic reflections (Cu Kα_1_ radiation, 2θ in °, rel. intensities: ss = very strong, s = strong, m = medium, mw = medium–weak, w = weak): 7.6 (w), 9.1 (ss), 9.4 (ss), 9.5 (*m*), 11.9 (*m*), 12.3 (*s*), 13.2 (mw), 13.5 (mw), 15.3 (w), 15.6 (*s*), 15.9 (mw), 16.7 (ss), 17.1 (w), 17.8 (w), 18.2 (mw), 18.5 (w), 18.7 (w), 18.9 (mw), 19.1 (w), 19.4 (*m*), 19.7 (*m*), 19.9 (mw), 20.5 (*m*), 21.5 (*m*), 22.1 (w), 22.3 (mw), 22.7 (mw), 23.6 (w), 23.9 (w), 24.1(w), 24.4(mw), 24.8(w), 26.1(w), 26.2 (w), 26.6 (w), 26.9(w), 27.2(w), 27.6(w), 28.1 (w), 28.4 (w), 28.9 (w), 29.6(w), 30.0(w), 30.1 (w), 31.3 (w), 31.6 (w), 32.1 (w), 33.7 (w), 34.3 (w), 36.2 (w), 36.4(w), 37.6 (w), 37.8 (w), 38.9(w), 39.3 (w).

### Refinement

The H atoms were positioned geometrically and treated as riding with C_primary_—H = 0.98 Å, C_secondary_—H = 0.97 Å, C_methyl_—H = 0.96 Å, O—H = 0.82 Å, *U*_iso_(*H*) = 1.2*U*_eq_(C_non-methyl_) and *U*_iso_(*H*) = 1.5*U*_eq_(C_methyl_,*O*). The H atoms at water molecule O7W were taken from a difference Fourier synthesis and were refined, using an O—H distance restraint of 0.84 (1) Å. The O and C atoms of the disordered DMSO solvate molecule were refined as anisotropic split atoms, using 20 restraints to bond distances and atomic displacement parameters. The occupancy factors refined to 0.482 (4) for atoms O15, C40, C41 and to 0.518 (4) for atoms O15', C40', C41'. The occupancy factor of the water molecule O8W refined to 0.432 (11). H atoms associated with the partially occupied water molecule (O8W) could not be located in difference Fourier maps and were not assigned.

### Crystal data

**Table:**

C_37_H_67_NO_13_·2C_2_H_6_SO·1.43H_2_O	*F*(000) = 997
*M**_r_* = 915.93	*D*_x_ = 1.218 Mg m^−^^3^
Monoclinic, *P*2_1_	Mo *K*α radiation, λ = 0.71073 Å
Hall symbol: P 2yb	Cell parameters from 6074 reflections
*a* = 11.1716 (7) Å	θ = 3–24°
*b* = 19.4025 (12) Å	µ = 0.17 mm^−^^1^
*c* = 12.0025 (7) Å	*T* = 296 K
β = 106.245 (1)°	Block, colourless
*V* = 2497.8 (3) Å^3^	0.55 × 0.42 × 0.32 mm
*Z* = 2	

### Data collection

**Table:**

Siemens SMART 1K CCD diffractometer	10520 independent reflections
Radiation source: normal-focus sealed tube	7109 reflections with *I* > 2σ(*I*)
Graphite monochromator	*R*_int_ = 0.037
ω scans	θ_max_ = 27.0°, θ_min_ = 2.1°
Absorption correction: multi-scan (*SADABS*; Sheldrick, 2000)	*h* = −14→14
*T*_min_ = 0.870, *T*_max_ = 0.947	*k* = −24→24
27453 measured reflections	*l* = −15→15

### Refinement

**Table:**

Refinement on *F*^2^	Secondary atom site location: difference Fourier map
Least-squares matrix: full	Hydrogen site location: inferred from neighbouring sites
*R*[*F*^2^ > 2σ(*F*^2^)] = 0.062	H atoms treated by a mixture of independent and constrained refinement
*wR*(*F*^2^) = 0.142	*w* = 1/[σ^2^(*F*_o_^2^) + (0.062*P*)^2^ + 0.61*P*] where *P* = (*F*_o_^2^ + 2*F*_c_^2^)/3
*S* = 1.04	(Δ/σ)_max_ < 0.001
10520 reflections	Δρ_max_ = 0.37 e Å^−^^3^
579 parameters	Δρ_min_ = −0.34 e Å^−^^3^
23 restraints	Absolute structure: Flack (1983), with 4965 Friedel pairs
Primary atom site location: structure-invariant direct methods	Flack parameter: 0.02 (9)

### Special details

**Table:**

Geometry. All e.s.d.'s (except the e.s.d. in the dihedral angle between two l.s. planes) are estimated using the full covariance matrix. The cell e.s.d.'s are taken into account individually in the estimation of e.s.d.'s in distances, angles and torsion angles; correlations between e.s.d.'s in cell parameters are only used when they are defined by crystal symmetry. An approximate (isotropic) treatment of cell e.s.d.'s is used for estimating e.s.d.'s involving l.s. planes.
Refinement. Refinement of *F*^2^ against ALL reflections. The weighted *R*-factor *wR* and goodness of fit *S* are based on *F*^2^, conventional *R*-factors *R* are based on *F*, with *F* set to zero for negative *F*^2^. The threshold expression of *F*^2^ > σ(*F*^2^) is used only for calculating *R*-factors(gt) *etc*. and is not relevant to the choice of reflections for refinement. *R*-factors based on *F*^2^ are statistically about twice as large as those based on *F*, and *R*-factors based on ALL data will be even larger.

### Fractional atomic coordinates and isotropic or equivalent isotropic displacement parameters (Å^2^)

**Table:**

	*x*	*y*	*z*	*U*_iso_*/*U*_eq_	Occ. (<1)
O1	0.9259 (2)	0.73051 (18)	0.7044 (2)	0.0678 (8)	
O3	0.89509 (19)	0.71189 (13)	1.06566 (17)	0.0416 (5)	
O5	0.66199 (19)	0.54287 (13)	0.93991 (17)	0.0379 (5)	
O6	0.9249 (2)	0.56635 (14)	0.8216 (2)	0.0498 (6)	
H6A	0.9900	0.5650	0.8743	0.075*	
O7W	0.1470 (3)	0.57984 (19)	1.0016 (2)	0.0695 (8)	
H7C	0.129 (4)	0.592 (3)	1.063 (2)	0.11 (2)*	
H7D	0.207 (3)	0.552 (2)	1.022 (3)	0.094 (18)*	
O8W	0.5845 (8)	0.9276 (5)	0.2582 (8)	0.106 (4)	0.432 (11)
O9	0.5978 (4)	0.47211 (19)	0.4648 (3)	0.0971 (11)	
O11	0.7227 (3)	0.63909 (15)	0.4476 (2)	0.0601 (7)	
H11B	0.6961	0.6645	0.3916	0.090*	
O12	0.5552 (3)	0.73381 (19)	0.3910 (2)	0.0767 (9)	
H12A	0.4817	0.7454	0.3714	0.115*	
O13	0.7336 (2)	0.77075 (14)	0.68886 (18)	0.0441 (5)	
O24	0.8552 (2)	0.73134 (15)	1.3084 (2)	0.0556 (6)	
O25	0.9804 (2)	0.62995 (16)	1.4629 (2)	0.0600 (7)	
H25B	0.9057	0.6380	1.4505	0.090*	
O26	1.0377 (2)	0.63220 (14)	1.17612 (19)	0.0493 (6)	
O30	0.7064 (2)	0.47251 (14)	1.0976 (2)	0.0511 (6)	
O34	0.4210 (2)	0.56850 (14)	0.9577 (2)	0.0517 (6)	
H34B	0.4199	0.5717	0.8893	0.078*	
N33	0.3193 (3)	0.46849 (16)	1.0750 (2)	0.0466 (7)	
C1	0.8498 (3)	0.75202 (17)	0.7486 (3)	0.0389 (7)	
C2	0.8725 (3)	0.76395 (17)	0.8779 (3)	0.0389 (7)	
H2A	0.8037	0.7917	0.8895	0.047*	
C3	0.8777 (3)	0.69572 (17)	0.9450 (2)	0.0335 (7)	
H3A	0.9491	0.6686	0.9375	0.040*	
C4	0.7580 (3)	0.65215 (17)	0.9018 (3)	0.0363 (7)	
H4A	0.7360	0.6524	0.8168	0.044*	
C5	0.7790 (3)	0.57594 (17)	0.9404 (3)	0.0345 (7)	
H5A	0.8368	0.5736	1.0185	0.041*	
C6	0.8284 (3)	0.52994 (18)	0.8564 (3)	0.0441 (8)	
C7	0.7203 (3)	0.5174 (2)	0.7452 (3)	0.0469 (8)	
H7A	0.6788	0.4750	0.7556	0.056*	
H7B	0.6605	0.5545	0.7385	0.056*	
C8	0.7540 (4)	0.51235 (19)	0.6293 (3)	0.0533 (9)	
H8A	0.8079	0.5515	0.6250	0.064*	
C9	0.6353 (4)	0.5193 (2)	0.5315 (3)	0.0599 (11)	
C10	0.5615 (3)	0.5868 (2)	0.5174 (3)	0.0555 (10)	
H10A	0.5242	0.5897	0.5821	0.067*	
C11	0.6505 (3)	0.6485 (2)	0.5281 (3)	0.0479 (9)	
H11A	0.7088	0.6463	0.6061	0.058*	
C12	0.5943 (3)	0.7213 (2)	0.5137 (3)	0.0515 (9)	
C13	0.7001 (3)	0.7736 (2)	0.5620 (3)	0.0478 (8)	
H13A	0.7728	0.7604	0.5363	0.057*	
C14	0.9918 (4)	0.8051 (2)	0.9199 (3)	0.0569 (10)	
H14A	0.9844	0.8471	0.8762	0.085*	
H14B	1.0603	0.7785	0.9093	0.085*	
H14C	1.0063	0.8158	1.0007	0.085*	
C15	0.6484 (3)	0.6862 (2)	0.9362 (4)	0.0573 (10)	
H15A	0.6401	0.7332	0.9105	0.086*	
H15B	0.6642	0.6847	1.0190	0.086*	
H15C	0.5727	0.6617	0.9003	0.086*	
C16	0.8815 (4)	0.46350 (19)	0.9167 (4)	0.0577 (10)	
H16A	0.9136	0.4359	0.8653	0.087*	
H16B	0.8169	0.4385	0.9379	0.087*	
H16C	0.9474	0.4740	0.9853	0.087*	
C17	0.8250 (5)	0.4457 (3)	0.6187 (5)	0.0940 (17)	
H17A	0.8444	0.4454	0.5457	0.141*	
H17B	0.7739	0.4065	0.6229	0.141*	
H17C	0.9008	0.4436	0.6808	0.141*	
C18	0.4532 (4)	0.5846 (3)	0.4040 (4)	0.0864 (15)	
H18A	0.4033	0.5444	0.4040	0.130*	
H18B	0.4866	0.5831	0.3385	0.130*	
H18C	0.4025	0.6251	0.3991	0.130*	
C19	0.4898 (4)	0.7309 (3)	0.5700 (4)	0.0748 (12)	
H19A	0.4251	0.6979	0.5386	0.112*	
H19B	0.4567	0.7767	0.5547	0.112*	
H19C	0.5215	0.7242	0.6522	0.112*	
C20	0.6678 (4)	0.8489 (2)	0.5289 (3)	0.0692 (12)	
H20A	0.6292	0.8517	0.4459	0.083*	
H20B	0.6076	0.8649	0.5677	0.083*	
C21	0.7804 (5)	0.8958 (2)	0.5608 (4)	0.0855 (15)	
H21A	0.7549	0.9423	0.5386	0.128*	
H21B	0.8395	0.8811	0.5210	0.128*	
H21C	0.8183	0.8939	0.6431	0.128*	
C22	1.0168 (3)	0.7020 (2)	1.1400 (3)	0.0445 (8)	
H22A	1.0756	0.7131	1.0954	0.053*	
C23	1.0425 (4)	0.7513 (2)	1.2420 (3)	0.0535 (9)	
H23A	1.0095	0.7962	1.2135	0.064*	
H23B	1.1320	0.7561	1.2734	0.064*	
C24	0.9880 (3)	0.7304 (2)	1.3406 (3)	0.0502 (9)	
C25	1.0223 (3)	0.6553 (2)	1.3694 (3)	0.0476 (9)	
H25A	1.1136	0.6529	1.3941	0.057*	
C26	0.9787 (3)	0.6105 (2)	1.2625 (3)	0.0473 (8)	
H26A	0.8881	0.6145	1.2312	0.057*	
C27	1.0414 (5)	0.7765 (3)	1.4463 (3)	0.0729 (12)	
H27A	1.0196	0.8236	1.4260	0.109*	
H27B	1.1305	0.7719	1.4711	0.109*	
H27C	1.0075	0.7629	1.5082	0.109*	
C28	0.7942 (5)	0.7927 (2)	1.2621 (4)	0.0775 (13)	
H28A	0.8396	0.8315	1.3027	0.116*	
H28B	0.7113	0.7928	1.2707	0.116*	
H28C	0.7900	0.7958	1.1813	0.116*	
C29	1.0136 (5)	0.5356 (2)	1.2872 (4)	0.0731 (12)	
H29A	0.9827	0.5092	1.2175	0.110*	
H29B	0.9776	0.5187	1.3457	0.110*	
H29C	1.1027	0.5314	1.3140	0.110*	
C30	0.6344 (3)	0.52944 (18)	1.0432 (3)	0.0379 (7)	
H30A	0.6512	0.5700	1.0939	0.046*	
C31	0.6781 (3)	0.4524 (2)	1.2038 (3)	0.0551 (10)	
H31A	0.6926	0.4917	1.2571	0.066*	
C32	0.5423 (3)	0.4309 (2)	1.1766 (3)	0.0571 (10)	
H32A	0.5225	0.4206	1.2486	0.069*	
H32B	0.5295	0.3893	1.1301	0.069*	
C33	0.4542 (3)	0.48677 (19)	1.1117 (3)	0.0414 (8)	
H33A	0.4631	0.5260	1.1647	0.050*	
C34	0.4951 (3)	0.51140 (18)	1.0086 (3)	0.0379 (7)	
H34A	0.4807	0.4741	0.9512	0.046*	
C35	0.7669 (4)	0.3950 (3)	1.2574 (4)	0.0831 (14)	
H35A	0.8512	0.4112	1.2729	0.125*	
H35B	0.7546	0.3567	1.2047	0.125*	
H35C	0.7515	0.3806	1.3286	0.125*	
C36	0.2757 (4)	0.4494 (2)	1.1756 (4)	0.0681 (12)	
H36A	0.1863	0.4466	1.1525	0.102*	
H36B	0.3022	0.4837	1.2352	0.102*	
H36C	0.3101	0.4055	1.2049	0.102*	
C37	0.2853 (4)	0.4155 (2)	0.9868 (3)	0.0574 (10)	
H37A	0.3259	0.3731	1.0169	0.086*	
H37B	0.3109	0.4293	0.9202	0.086*	
H37C	0.1966	0.4090	0.9648	0.086*	
S1	0.25601 (10)	0.59971 (10)	0.65505 (9)	0.0772 (4)	
O14	0.3849 (3)	0.5796 (2)	0.7224 (3)	0.0901 (11)	
C38	0.2103 (6)	0.6649 (3)	0.7334 (6)	0.118 (2)	
H38A	0.2281	0.6514	0.8133	0.177*	
H38B	0.1224	0.6729	0.7026	0.177*	
H38C	0.2550	0.7063	0.7276	0.177*	
C39	0.1537 (5)	0.5351 (4)	0.6804 (5)	0.1078 (19)	
H39A	0.1596	0.4947	0.6361	0.162*	
H39B	0.0696	0.5520	0.6575	0.162*	
H39C	0.1767	0.5237	0.7615	0.162*	
S2	0.32316 (11)	0.78019 (6)	0.13850 (11)	0.0831 (4)	0.482 (4)
O15	0.3312 (7)	0.7602 (4)	0.2610 (4)	0.0988 (12)	0.482 (4)
C40	0.3945 (18)	0.7122 (7)	0.0884 (13)	0.129 (3)	0.482 (4)
H40D	0.3430	0.6719	0.0804	0.193*	0.482 (4)
H40E	0.4741	0.7031	0.1426	0.193*	0.482 (4)
H40F	0.4060	0.7239	0.0143	0.193*	0.482 (4)
C41	0.4354 (12)	0.8434 (6)	0.1485 (12)	0.126 (3)	0.482 (4)
H41A	0.4095	0.8850	0.1784	0.189*	0.482 (4)
H41B	0.4456	0.8521	0.0729	0.189*	0.482 (4)
H41C	0.5131	0.8282	0.1997	0.189*	0.482 (4)
S2'	0.32316 (11)	0.78019 (6)	0.13850 (11)	0.0831 (4)	0.518 (4)
O15'	0.3821 (7)	0.8140 (3)	0.2530 (4)	0.0988 (12)	0.518 (4)
C40'	0.3841 (18)	0.6976 (5)	0.1450 (13)	0.129 (3)	0.518 (4)
H40A	0.3503	0.6696	0.1947	0.193*	0.518 (4)
H40B	0.4732	0.6995	0.1752	0.193*	0.518 (4)
H40C	0.3626	0.6780	0.0685	0.193*	0.518 (4)
C41'	0.4084 (12)	0.8139 (7)	0.0506 (10)	0.126 (3)	0.518 (4)
H41D	0.3887	0.8619	0.0367	0.189*	0.518 (4)
H41E	0.3878	0.7896	−0.0219	0.189*	0.518 (4)
H41F	0.4958	0.8089	0.0885	0.189*	0.518 (4)

### Atomic displacement parameters (Å^2^)

**Table:**

	*U*^11^	*U*^22^	*U*^33^	*U*^12^	*U*^13^	*U*^23^
O1	0.0607 (16)	0.100 (2)	0.0466 (14)	0.0232 (16)	0.0213 (13)	0.0065 (15)
O3	0.0416 (12)	0.0527 (14)	0.0337 (11)	0.0013 (10)	0.0155 (10)	0.0010 (10)
O5	0.0376 (12)	0.0434 (12)	0.0368 (11)	−0.0056 (10)	0.0174 (9)	0.0028 (10)
O6	0.0447 (13)	0.0531 (14)	0.0593 (15)	−0.0022 (12)	0.0272 (11)	−0.0008 (12)
O7W	0.0553 (17)	0.089 (2)	0.0660 (18)	0.0140 (16)	0.0195 (14)	0.0020 (17)
O8W	0.098 (7)	0.107 (7)	0.108 (7)	0.008 (5)	0.018 (5)	0.035 (5)
O9	0.133 (3)	0.071 (2)	0.085 (2)	−0.027 (2)	0.026 (2)	−0.0360 (19)
O11	0.0719 (18)	0.0600 (17)	0.0573 (16)	−0.0040 (13)	0.0328 (14)	0.0000 (13)
O12	0.079 (2)	0.096 (2)	0.0426 (14)	0.0130 (19)	−0.0045 (13)	−0.0086 (15)
O13	0.0442 (13)	0.0525 (14)	0.0348 (11)	0.0021 (11)	0.0095 (10)	−0.0015 (10)
O24	0.0551 (15)	0.0602 (16)	0.0537 (14)	0.0110 (13)	0.0187 (12)	0.0153 (13)
O25	0.0606 (16)	0.0774 (19)	0.0468 (14)	0.0079 (14)	0.0230 (12)	0.0203 (13)
O26	0.0482 (13)	0.0596 (16)	0.0418 (13)	0.0092 (11)	0.0155 (11)	0.0054 (11)
O30	0.0460 (14)	0.0614 (16)	0.0490 (14)	0.0072 (12)	0.0185 (11)	0.0173 (12)
O34	0.0453 (13)	0.0538 (15)	0.0624 (15)	0.0109 (11)	0.0254 (12)	0.0157 (12)
N33	0.0462 (16)	0.0517 (18)	0.0483 (17)	−0.0118 (14)	0.0235 (13)	−0.0027 (14)
C1	0.0444 (19)	0.0345 (18)	0.0403 (17)	−0.0037 (15)	0.0162 (15)	0.0054 (14)
C2	0.0419 (17)	0.0390 (18)	0.0359 (16)	−0.0019 (14)	0.0109 (14)	−0.0022 (14)
C3	0.0350 (16)	0.0338 (16)	0.0323 (15)	0.0028 (13)	0.0106 (13)	0.0019 (13)
C4	0.0318 (16)	0.0384 (19)	0.0396 (17)	0.0003 (13)	0.0115 (13)	0.0022 (14)
C5	0.0293 (15)	0.0380 (17)	0.0377 (16)	−0.0026 (13)	0.0119 (13)	0.0056 (14)
C6	0.0425 (19)	0.0395 (19)	0.056 (2)	−0.0014 (15)	0.0229 (16)	0.0039 (16)
C7	0.053 (2)	0.046 (2)	0.048 (2)	−0.0054 (16)	0.0236 (16)	−0.0068 (16)
C8	0.075 (3)	0.038 (2)	0.058 (2)	−0.0027 (18)	0.036 (2)	−0.0088 (17)
C9	0.083 (3)	0.054 (3)	0.051 (2)	−0.020 (2)	0.032 (2)	−0.015 (2)
C10	0.056 (2)	0.071 (3)	0.0400 (18)	−0.017 (2)	0.0141 (16)	−0.0113 (18)
C11	0.0454 (19)	0.061 (2)	0.0383 (19)	−0.0096 (17)	0.0137 (15)	−0.0058 (17)
C12	0.048 (2)	0.060 (2)	0.0412 (19)	−0.0007 (18)	0.0044 (16)	−0.0028 (17)
C13	0.059 (2)	0.048 (2)	0.0343 (17)	−0.0002 (17)	0.0096 (15)	0.0023 (15)
C14	0.063 (2)	0.053 (2)	0.052 (2)	−0.0158 (19)	0.0111 (18)	0.0087 (18)
C15	0.040 (2)	0.042 (2)	0.096 (3)	0.0038 (16)	0.028 (2)	0.006 (2)
C16	0.062 (2)	0.043 (2)	0.073 (3)	0.0062 (18)	0.027 (2)	0.0029 (19)
C17	0.127 (4)	0.081 (3)	0.093 (4)	0.023 (3)	0.062 (3)	−0.019 (3)
C18	0.082 (3)	0.104 (4)	0.061 (3)	−0.033 (3)	−0.001 (2)	−0.017 (3)
C19	0.049 (2)	0.092 (3)	0.080 (3)	0.004 (2)	0.014 (2)	−0.011 (3)
C20	0.097 (3)	0.055 (2)	0.047 (2)	0.000 (2)	0.006 (2)	0.0005 (19)
C21	0.144 (5)	0.050 (3)	0.072 (3)	−0.011 (3)	0.044 (3)	−0.002 (2)
C22	0.0383 (18)	0.058 (2)	0.0389 (17)	−0.0050 (16)	0.0140 (15)	0.0011 (16)
C23	0.058 (2)	0.063 (2)	0.0410 (19)	−0.0133 (19)	0.0151 (16)	−0.0005 (17)
C24	0.060 (2)	0.055 (2)	0.0375 (18)	−0.0081 (18)	0.0171 (16)	−0.0008 (16)
C25	0.0391 (18)	0.069 (2)	0.0361 (17)	0.0025 (17)	0.0121 (14)	0.0079 (17)
C26	0.0414 (18)	0.056 (2)	0.0440 (19)	0.0052 (17)	0.0105 (15)	0.0089 (17)
C27	0.104 (3)	0.075 (3)	0.045 (2)	−0.019 (3)	0.028 (2)	−0.014 (2)
C28	0.098 (3)	0.067 (3)	0.068 (3)	0.030 (3)	0.025 (3)	0.014 (2)
C29	0.107 (4)	0.049 (2)	0.064 (3)	0.015 (2)	0.025 (2)	0.010 (2)
C30	0.0440 (18)	0.0349 (18)	0.0380 (17)	−0.0009 (14)	0.0166 (14)	0.0002 (14)
C31	0.053 (2)	0.069 (3)	0.045 (2)	−0.0018 (19)	0.0162 (17)	0.0171 (18)
C32	0.063 (2)	0.062 (2)	0.052 (2)	−0.008 (2)	0.0256 (19)	0.0126 (19)
C33	0.0416 (18)	0.048 (2)	0.0362 (17)	−0.0073 (15)	0.0142 (14)	−0.0032 (15)
C34	0.0397 (17)	0.0386 (18)	0.0393 (17)	−0.0004 (15)	0.0174 (14)	0.0006 (15)
C35	0.074 (3)	0.094 (4)	0.078 (3)	0.005 (3)	0.016 (3)	0.041 (3)
C36	0.072 (3)	0.082 (3)	0.065 (3)	−0.024 (2)	0.043 (2)	−0.012 (2)
C37	0.055 (2)	0.055 (2)	0.063 (2)	−0.0121 (18)	0.0181 (19)	−0.0080 (19)
S1	0.0550 (6)	0.1259 (11)	0.0512 (6)	0.0139 (7)	0.0158 (5)	0.0244 (6)
O14	0.0610 (18)	0.141 (3)	0.0719 (19)	0.027 (2)	0.0256 (15)	0.024 (2)
C38	0.112 (5)	0.118 (5)	0.116 (5)	0.045 (4)	0.019 (4)	0.007 (4)
C39	0.086 (4)	0.140 (5)	0.096 (4)	0.000 (4)	0.025 (3)	0.023 (4)
S2	0.0556 (6)	0.1101 (10)	0.0765 (7)	0.0036 (6)	0.0067 (5)	0.0220 (7)
O15	0.104 (4)	0.108 (3)	0.0781 (11)	−0.003 (2)	0.014 (2)	0.0190 (15)
C40	0.160 (7)	0.121 (3)	0.103 (8)	0.032 (5)	0.032 (7)	0.010 (2)
C41	0.136 (7)	0.148 (5)	0.106 (4)	−0.052 (4)	0.054 (4)	−0.014 (3)
S2'	0.0556 (6)	0.1101 (10)	0.0765 (7)	0.0036 (6)	0.0067 (5)	0.0220 (7)
O15'	0.104 (4)	0.108 (3)	0.0781 (11)	−0.003 (2)	0.014 (2)	0.0190 (15)
C40'	0.160 (7)	0.121 (3)	0.103 (8)	0.032 (5)	0.032 (7)	0.010 (2)
C41'	0.136 (7)	0.148 (5)	0.106 (4)	−0.052 (4)	0.054 (4)	−0.014 (3)

### Geometric parameters (Å, º)

**Table:**

O1—C1	1.197 (4)	C19—H19A	0.9600
O3—C22	1.416 (4)	C19—H19B	0.9600
O3—C3	1.440 (4)	C19—H19C	0.9600
O5—C30	1.383 (3)	C20—C21	1.513 (7)
O5—C5	1.454 (3)	C20—H20A	0.9700
O6—C6	1.445 (4)	C20—H20B	0.9700
O6—H6A	0.8200	C21—H21A	0.9600
O7W—H7C	0.845 (10)	C21—H21B	0.9600
O7W—H7D	0.841 (10)	C21—H21C	0.9600
O9—C9	1.210 (5)	C22—C23	1.517 (5)
O11—C11	1.434 (4)	C22—H22A	0.9800
O11—H11B	0.8200	C23—C24	1.529 (5)
O12—C12	1.436 (4)	C23—H23A	0.9700
O12—H12A	0.8200	C23—H23B	0.9700
O13—C1	1.345 (4)	C24—C25	1.523 (5)
O13—C13	1.464 (4)	C24—C27	1.530 (5)
O24—C28	1.407 (5)	C25—C26	1.512 (5)
O24—C24	1.425 (4)	C25—H25A	0.9800
O25—C25	1.419 (4)	C26—C29	1.513 (5)
O25—H25B	0.8200	C26—H26A	0.9800
O26—C22	1.421 (4)	C27—H27A	0.9600
O26—C26	1.438 (4)	C27—H27B	0.9600
O30—C30	1.414 (4)	C27—H27C	0.9600
O30—C31	1.450 (4)	C28—H28A	0.9600
O34—C34	1.415 (4)	C28—H28B	0.9600
O34—H34B	0.8200	C28—H28C	0.9600
N33—C37	1.448 (5)	C29—H29A	0.9600
N33—C36	1.470 (4)	C29—H29B	0.9600
N33—C33	1.490 (4)	C29—H29C	0.9600
C1—C2	1.519 (4)	C30—C34	1.534 (4)
C2—C14	1.514 (5)	C30—H30A	0.9800
C2—C3	1.542 (4)	C31—C35	1.511 (6)
C2—H2A	0.9800	C31—C32	1.518 (5)
C3—C4	1.544 (4)	C31—H31A	0.9800
C3—H3A	0.9800	C32—C33	1.523 (5)
C4—C15	1.546 (5)	C32—H32A	0.9700
C4—C5	1.548 (4)	C32—H32B	0.9700
C4—H4A	0.9800	C33—C34	1.511 (4)
C5—C6	1.558 (4)	C33—H33A	0.9800
C5—H5A	0.9800	C34—H34A	0.9800
C6—C16	1.516 (5)	C35—H35A	0.9600
C6—C7	1.548 (5)	C35—H35B	0.9600
C7—C8	1.542 (5)	C35—H35C	0.9600
C7—H7A	0.9700	C36—H36A	0.9600
C7—H7B	0.9700	C36—H36B	0.9600
C8—C9	1.512 (6)	C36—H36C	0.9600
C8—C17	1.542 (6)	C37—H37A	0.9600
C8—H8A	0.9800	C37—H37B	0.9600
C9—C10	1.532 (6)	C37—H37C	0.9600
C10—C11	1.537 (5)	S1—O14	1.493 (3)
C10—C18	1.549 (5)	S1—C38	1.735 (6)
C10—H10A	0.9800	S1—C39	1.779 (6)
C11—C12	1.536 (6)	C38—H38A	0.9600
C11—H11A	0.9800	C38—H38B	0.9600
C12—C19	1.514 (5)	C38—H38C	0.9600
C12—C13	1.544 (5)	C39—H39A	0.9600
C13—C20	1.530 (6)	C39—H39B	0.9600
C13—H13A	0.9800	C39—H39C	0.9600
C14—H14A	0.9600	S2—O15	1.498 (5)
C14—H14B	0.9600	S2—C41	1.734 (6)
C14—H14C	0.9600	S2—C40	1.734 (6)
C15—H15A	0.9600	C40—H40D	0.9600
C15—H15B	0.9600	C40—H40E	0.9600
C15—H15C	0.9600	C40—H40F	0.9600
C16—H16A	0.9600	C41—H41A	0.9600
C16—H16B	0.9600	C41—H41B	0.9600
C16—H16C	0.9600	C41—H41C	0.9600
C17—H17A	0.9600	C40'—H40A	0.9600
C17—H17B	0.9600	C40'—H40B	0.9600
C17—H17C	0.9600	C40'—H40C	0.9600
C18—H18A	0.9600	C41'—H41D	0.9600
C18—H18B	0.9600	C41'—H41E	0.9600
C18—H18C	0.9600	C41'—H41F	0.9600
			
C22—O3—C3	116.0 (2)	H21B—C21—H21C	109.5
C30—O5—C5	120.3 (2)	O3—C22—O26	111.6 (3)
C6—O6—H6A	109.5	O3—C22—C23	110.7 (3)
H7C—O7W—H7D	106 (4)	O26—C22—C23	112.1 (3)
C11—O11—H11B	109.5	O3—C22—H22A	107.4
C12—O12—H12A	109.5	O26—C22—H22A	107.4
C1—O13—C13	119.5 (2)	C23—C22—H22A	107.4
C28—O24—C24	118.0 (3)	C22—C23—C24	115.6 (3)
C25—O25—H25B	109.5	C22—C23—H23A	108.4
C22—O26—C26	115.4 (3)	C24—C23—H23A	108.4
C30—O30—C31	112.4 (2)	C22—C23—H23B	108.4
C34—O34—H34B	109.5	C24—C23—H23B	108.4
C37—N33—C36	110.0 (3)	H23A—C23—H23B	107.4
C37—N33—C33	114.9 (3)	O24—C24—C25	104.5 (3)
C36—N33—C33	111.0 (3)	O24—C24—C23	113.3 (3)
O1—C1—O13	123.8 (3)	C25—C24—C23	107.4 (3)
O1—C1—C2	124.8 (3)	O24—C24—C27	110.7 (3)
O13—C1—C2	111.4 (3)	C25—C24—C27	110.9 (3)
C14—C2—C1	107.5 (3)	C23—C24—C27	109.9 (3)
C14—C2—C3	112.2 (3)	O25—C25—C26	111.9 (3)
C1—C2—C3	112.0 (2)	O25—C25—C24	113.2 (3)
C14—C2—H2A	108.4	C26—C25—C24	110.9 (3)
C1—C2—H2A	108.4	O25—C25—H25A	106.8
C3—C2—H2A	108.4	C26—C25—H25A	106.8
O3—C3—C2	108.2 (2)	C24—C25—H25A	106.8
O3—C3—C4	108.5 (2)	O26—C26—C25	109.9 (3)
C2—C3—C4	113.1 (2)	O26—C26—C29	106.1 (3)
O3—C3—H3A	109.0	C25—C26—C29	112.4 (3)
C2—C3—H3A	109.0	O26—C26—H26A	109.5
C4—C3—H3A	109.0	C25—C26—H26A	109.5
C3—C4—C15	110.7 (3)	C29—C26—H26A	109.5
C3—C4—C5	112.2 (2)	C24—C27—H27A	109.5
C15—C4—C5	113.2 (3)	C24—C27—H27B	109.5
C3—C4—H4A	106.8	H27A—C27—H27B	109.5
C15—C4—H4A	106.8	C24—C27—H27C	109.5
C5—C4—H4A	106.8	H27A—C27—H27C	109.5
O5—C5—C4	111.1 (2)	H27B—C27—H27C	109.5
O5—C5—C6	103.0 (2)	O24—C28—H28A	109.5
C4—C5—C6	113.9 (2)	O24—C28—H28B	109.5
O5—C5—H5A	109.5	H28A—C28—H28B	109.5
C4—C5—H5A	109.5	O24—C28—H28C	109.5
C6—C5—H5A	109.5	H28A—C28—H28C	109.5
O6—C6—C16	109.3 (3)	H28B—C28—H28C	109.5
O6—C6—C7	107.0 (3)	C26—C29—H29A	109.5
C16—C6—C7	112.6 (3)	C26—C29—H29B	109.5
O6—C6—C5	109.6 (3)	H29A—C29—H29B	109.5
C16—C6—C5	109.9 (3)	C26—C29—H29C	109.5
C7—C6—C5	108.4 (3)	H29A—C29—H29C	109.5
C8—C7—C6	117.4 (3)	H29B—C29—H29C	109.5
C8—C7—H7A	108.0	O5—C30—O30	108.7 (2)
C6—C7—H7A	108.0	O5—C30—C34	105.3 (2)
C8—C7—H7B	108.0	O30—C30—C34	110.5 (2)
C6—C7—H7B	108.0	O5—C30—H30A	110.7
H7A—C7—H7B	107.2	O30—C30—H30A	110.7
C9—C8—C17	111.7 (3)	C34—C30—H30A	110.7
C9—C8—C7	108.2 (3)	O30—C31—C35	107.1 (3)
C17—C8—C7	112.9 (3)	O30—C31—C32	109.6 (3)
C9—C8—H8A	107.9	C35—C31—C32	112.8 (3)
C17—C8—H8A	107.9	O30—C31—H31A	109.1
C7—C8—H8A	107.9	C35—C31—H31A	109.1
O9—C9—C8	120.9 (4)	C32—C31—H31A	109.1
O9—C9—C10	119.8 (4)	C31—C32—C33	112.3 (3)
C8—C9—C10	119.3 (3)	C31—C32—H32A	109.2
C9—C10—C11	109.9 (3)	C33—C32—H32A	109.2
C9—C10—C18	109.7 (3)	C31—C32—H32B	109.2
C11—C10—C18	115.0 (3)	C33—C32—H32B	109.2
C9—C10—H10A	107.3	H32A—C32—H32B	107.9
C11—C10—H10A	107.3	N33—C33—C34	110.3 (3)
C18—C10—H10A	107.3	N33—C33—C32	115.9 (3)
O11—C11—C12	109.3 (3)	C34—C33—C32	110.1 (3)
O11—C11—C10	108.5 (3)	N33—C33—H33A	106.7
C12—C11—C10	118.2 (3)	C34—C33—H33A	106.7
O11—C11—H11A	106.8	C32—C33—H33A	106.7
C12—C11—H11A	106.8	O34—C34—C33	109.0 (3)
C10—C11—H11A	106.8	O34—C34—C30	111.4 (3)
O12—C12—C19	112.7 (3)	C33—C34—C30	111.5 (3)
O12—C12—C11	105.6 (3)	O34—C34—H34A	108.3
C19—C12—C11	113.9 (3)	C33—C34—H34A	108.3
O12—C12—C13	104.8 (3)	C30—C34—H34A	108.3
C19—C12—C13	110.9 (3)	C31—C35—H35A	109.5
C11—C12—C13	108.5 (3)	C31—C35—H35B	109.5
O13—C13—C20	106.2 (3)	H35A—C35—H35B	109.5
O13—C13—C12	108.1 (3)	C31—C35—H35C	109.5
C20—C13—C12	115.5 (3)	H35A—C35—H35C	109.5
O13—C13—H13A	108.9	H35B—C35—H35C	109.5
C20—C13—H13A	108.9	N33—C36—H36A	109.5
C12—C13—H13A	108.9	N33—C36—H36B	109.5
C2—C14—H14A	109.5	H36A—C36—H36B	109.5
C2—C14—H14B	109.5	N33—C36—H36C	109.5
H14A—C14—H14B	109.5	H36A—C36—H36C	109.5
C2—C14—H14C	109.5	H36B—C36—H36C	109.5
H14A—C14—H14C	109.5	N33—C37—H37A	109.5
H14B—C14—H14C	109.5	N33—C37—H37B	109.5
C4—C15—H15A	109.5	H37A—C37—H37B	109.5
C4—C15—H15B	109.5	N33—C37—H37C	109.5
H15A—C15—H15B	109.5	H37A—C37—H37C	109.5
C4—C15—H15C	109.5	H37B—C37—H37C	109.5
H15A—C15—H15C	109.5	O14—S1—C38	106.4 (3)
H15B—C15—H15C	109.5	O14—S1—C39	107.0 (2)
C6—C16—H16A	109.5	C38—S1—C39	96.9 (3)
C6—C16—H16B	109.5	S1—C38—H38A	109.5
H16A—C16—H16B	109.5	S1—C38—H38B	109.5
C6—C16—H16C	109.5	H38A—C38—H38B	109.5
H16A—C16—H16C	109.5	S1—C38—H38C	109.5
H16B—C16—H16C	109.5	H38A—C38—H38C	109.5
C8—C17—H17A	109.5	H38B—C38—H38C	109.5
C8—C17—H17B	109.5	S1—C39—H39A	109.5
H17A—C17—H17B	109.5	S1—C39—H39B	109.5
C8—C17—H17C	109.5	H39A—C39—H39B	109.5
H17A—C17—H17C	109.5	S1—C39—H39C	109.5
H17B—C17—H17C	109.5	H39A—C39—H39C	109.5
C10—C18—H18A	109.5	H39B—C39—H39C	109.5
C10—C18—H18B	109.5	O15—S2—C41	105.7 (5)
H18A—C18—H18B	109.5	O15—S2—C40	103.7 (6)
C10—C18—H18C	109.5	C41—S2—C40	99.7 (9)
H18A—C18—H18C	109.5	S2—C40—H40D	109.5
H18B—C18—H18C	109.5	S2—C40—H40E	109.5
C12—C19—H19A	109.5	H40D—C40—H40E	109.5
C12—C19—H19B	109.5	S2—C40—H40F	109.5
H19A—C19—H19B	109.5	H40D—C40—H40F	109.5
C12—C19—H19C	109.5	H40E—C40—H40F	109.5
H19A—C19—H19C	109.5	S2—C41—H41A	109.5
H19B—C19—H19C	109.5	S2—C41—H41B	109.5
C21—C20—C13	112.9 (4)	H41A—C41—H41B	109.5
C21—C20—H20A	109.0	S2—C41—H41C	109.5
C13—C20—H20A	109.0	H41A—C41—H41C	109.5
C21—C20—H20B	109.0	H41B—C41—H41C	109.5
C13—C20—H20B	109.0	H40A—C40'—H40B	109.5
H20A—C20—H20B	107.8	H40A—C40'—H40C	109.5
C20—C21—H21A	109.5	H40B—C40'—H40C	109.5
C20—C21—H21B	109.5	H41D—C41'—H41E	109.5
H21A—C21—H21B	109.5	H41D—C41'—H41F	109.5
C20—C21—H21C	109.5	H41E—C41'—H41F	109.5
H21A—C21—H21C	109.5		
			
C13—O13—C1—O1	−7.9 (5)	C19—C12—C13—O13	50.4 (4)
C13—O13—C1—C2	170.3 (3)	C11—C12—C13—O13	−75.4 (3)
O1—C1—C2—C14	49.6 (4)	O12—C12—C13—C20	53.5 (4)
O13—C1—C2—C14	−128.6 (3)	C19—C12—C13—C20	−68.4 (4)
O1—C1—C2—C3	−74.1 (4)	C11—C12—C13—C20	165.8 (3)
O13—C1—C2—C3	107.8 (3)	O13—C13—C20—C21	70.1 (4)
C22—O3—C3—C2	−103.4 (3)	C12—C13—C20—C21	−170.1 (3)
C22—O3—C3—C4	133.6 (3)	C3—O3—C22—O26	−83.3 (3)
C14—C2—C3—O3	61.1 (3)	C3—O3—C22—C23	151.0 (3)
C1—C2—C3—O3	−178.0 (2)	C26—O26—C22—O3	−75.0 (3)
C14—C2—C3—C4	−178.7 (3)	C26—O26—C22—C23	49.9 (4)
C1—C2—C3—C4	−57.8 (3)	O3—C22—C23—C24	79.8 (4)
O3—C3—C4—C15	49.2 (3)	O26—C22—C23—C24	−45.6 (4)
C2—C3—C4—C15	−70.8 (3)	C28—O24—C24—C25	−171.9 (3)
O3—C3—C4—C5	−78.2 (3)	C28—O24—C24—C23	−55.2 (4)
C2—C3—C4—C5	161.7 (2)	C28—O24—C24—C27	68.7 (4)
C30—O5—C5—C4	−105.4 (3)	C22—C23—C24—O24	−66.5 (4)
C30—O5—C5—C6	132.3 (3)	C22—C23—C24—C25	48.4 (4)
C3—C4—C5—O5	157.8 (2)	C22—C23—C24—C27	169.1 (3)
C15—C4—C5—O5	31.6 (4)	O24—C24—C25—O25	−61.2 (3)
C3—C4—C5—C6	−86.4 (3)	C23—C24—C25—O25	178.1 (3)
C15—C4—C5—C6	147.4 (3)	C27—C24—C25—O25	58.0 (4)
O5—C5—C6—O6	163.7 (2)	O24—C24—C25—C26	65.4 (3)
C4—C5—C6—O6	43.3 (3)	C23—C24—C25—C26	−55.2 (4)
O5—C5—C6—C16	−76.2 (3)	C27—C24—C25—C26	−175.3 (3)
C4—C5—C6—C16	163.4 (3)	C22—O26—C26—C25	−58.2 (3)
O5—C5—C6—C7	47.3 (3)	C22—O26—C26—C29	−180.0 (3)
C4—C5—C6—C7	−73.1 (3)	O25—C25—C26—O26	−171.7 (3)
O6—C6—C7—C8	27.4 (4)	C24—C25—C26—O26	60.9 (4)
C16—C6—C7—C8	−92.7 (4)	O25—C25—C26—C29	−53.9 (4)
C5—C6—C7—C8	145.5 (3)	C24—C25—C26—C29	178.8 (3)
C6—C7—C8—C9	−165.1 (3)	C5—O5—C30—O30	−74.8 (3)
C6—C7—C8—C17	70.7 (5)	C5—O5—C30—C34	166.8 (2)
C17—C8—C9—O9	9.0 (5)	C31—O30—C30—O5	−176.7 (3)
C7—C8—C9—O9	−115.9 (4)	C31—O30—C30—C34	−61.7 (3)
C17—C8—C9—C10	−171.5 (3)	C30—O30—C31—C35	−176.0 (3)
C7—C8—C9—C10	63.6 (4)	C30—O30—C31—C32	61.3 (4)
O9—C9—C10—C11	−134.4 (4)	O30—C31—C32—C33	−55.0 (4)
C8—C9—C10—C11	46.0 (4)	C35—C31—C32—C33	−174.2 (3)
O9—C9—C10—C18	−6.9 (5)	C37—N33—C33—C34	58.7 (4)
C8—C9—C10—C18	173.5 (3)	C36—N33—C33—C34	−175.7 (3)
C9—C10—C11—O11	53.6 (4)	C37—N33—C33—C32	−67.3 (4)
C18—C10—C11—O11	−70.9 (4)	C36—N33—C33—C32	58.3 (4)
C9—C10—C11—C12	178.6 (3)	C31—C32—C33—N33	176.4 (3)
C18—C10—C11—C12	54.2 (4)	C31—C32—C33—C34	50.4 (4)
O11—C11—C12—O12	40.4 (4)	N33—C33—C34—O34	57.6 (3)
C10—C11—C12—O12	−84.3 (4)	C32—C33—C34—O34	−173.3 (3)
O11—C11—C12—C19	164.5 (3)	N33—C33—C34—C30	−179.0 (3)
C10—C11—C12—C19	39.9 (4)	C32—C33—C34—C30	−49.9 (4)
O11—C11—C12—C13	−71.4 (3)	O5—C30—C34—O34	−65.0 (3)
C10—C11—C12—C13	163.9 (3)	O30—C30—C34—O34	177.9 (2)
C1—O13—C13—C20	−117.1 (3)	O5—C30—C34—C33	173.0 (3)
C1—O13—C13—C12	118.3 (3)	O30—C30—C34—C33	55.8 (3)
O12—C12—C13—O13	172.3 (3)		

### Hydrogen-bond geometry (Å, º)

**Table:**

*D*—H···*A*	*D*—H	H···*A*	*D*···*A*	*D*—H···*A*
O6—H6*A*···O7*W*^i^	0.82	2.00	2.810 (4)	170
O7*W*—H7*C*···O26^ii^	0.85 (1)	2.07 (2)	2.888 (4)	163 (4)
O7*W*—H7*D*···N33	0.84 (1)	2.04 (2)	2.863 (4)	166 (5)
O11—H11*B*···O12	0.82	2.07	2.575 (4)	120
O12—H12*A*···O15	0.82	1.85	2.602 (8)	152
O12—H12*A*···O15′	0.82	2.04	2.667 (7)	134
O25—H25*B*···O11^iii^	0.82	2.04	2.838 (4)	166
O34—H34*B*···O14	0.82	1.94	2.747 (4)	170

Symmetry codes: (i) *x*+1, *y*, *z*; (ii) *x*−1, *y*, *z*; (iii) *x*, *y*, *z*+1.
